# Computational Design of a pH Stable Enzyme: Understanding Molecular Mechanism of Penicillin Acylase's Adaptation to Alkaline Conditions

**DOI:** 10.1371/journal.pone.0100643

**Published:** 2014-06-24

**Authors:** Dmitry Suplatov, Nikolay Panin, Evgeny Kirilin, Tatyana Shcherbakova, Pavel Kudryavtsev, Vytas Švedas

**Affiliations:** Lomonosov Moscow State University, Belozersky Institute of Physicochemical Biology and Faculty of Bioengineering and Bioinformatics, Moscow, Russia; Russian Academy of Sciences, Institute for Biological Instrumentation, Russian Federation

## Abstract

Protein stability provides advantageous development of novel properties and can be crucial in affording tolerance to mutations that introduce functionally preferential phenotypes. Consequently, understanding the determining factors for protein stability is important for the study of structure-function relationship and design of novel protein functions. Thermal stability has been extensively studied in connection with practical application of biocatalysts. However, little work has been done to explore the mechanism of pH-dependent inactivation. In this study, bioinformatic analysis of the Ntn-hydrolase superfamily was performed to identify functionally important subfamily-specific positions in protein structures. Furthermore, the involvement of these positions in pH-induced inactivation was studied. The conformational mobility of penicillin acylase in *Escherichia coli* was analyzed through molecular modeling in neutral and alkaline conditions. Two functionally important subfamily-specific residues, Gluβ482 and Aspβ484, were found. Ionization of these residues at alkaline pH promoted the collapse of a buried network of stabilizing interactions that consequently disrupted the functional protein conformation. The subfamily-specific position Aspβ484 was selected as a hotspot for mutation to engineer enzyme variant tolerant to alkaline medium. The corresponding Dβ484N mutant was produced and showed 9-fold increase in stability at alkaline conditions. Bioinformatic analysis of subfamily-specific positions can be further explored to study mechanisms of protein inactivation and to design more stable variants for the engineering of homologous Ntn-hydrolases with improved catalytic properties.

## Introduction

To perform their functions, most proteins form compact native structures that are stabilized by complex networks of covalent bonds, non-covalent hydrophobic, electrostatic, van der Waals interactions and hydrogen bonds [1]–[3]. Structural stability, therefore, is largely necessary for the maintenance of functional conformations under adverse environmental conditions (temperature, pressure, pH, presence of solvents, and salts, etc.). Stability is a fundamental property that not only affects the structure and function of macromolecules but also determines biological fitness. Retention of the native fold is, in general, a prerequisite for the evolution of new functions [4], [5]. Proteins that better tolerate functionally beneficial but destabilizing mutations have a higher chance to survive the selection pressure [6]. Consequently, extra stability provides a strong advantage in evolution and promotes evolvability [7], [8]. Thus, exploring the mechanisms of protein stability appears to be important not only for studying enzyme evolution and understanding structure-function relationship, but also for the engineering of novel enzymes.

Last century has witnessed a rapid emergence of biocatalysis and its use in the adaptation of enzymes to new industrial applications for which they have not been evolved [9]. The unique catalytic functions of enzymes are determined by their complex three-dimensional structures. In particular, the organization of the active site residues must satisfy certain structural constraints, which can result in poor stability [10], [11]. Consequently, stability emerged as a major limitation to the use of enzymes in non-natural environments [12]. Owing primarily to industrial needs, enzymes were extensively studied to understand the tradeoff between activity and stability and to engineer more tolerant variants [13]–[17]. These studies focused mainly on the thermal stability of the biocatalysts as many industrial processes involve reactions at elevated temperatures for improved productivity and for exclusion of microbial contamination.

Penicillin acylases (EC 3.5.1.11) are a group of enzymes mainly known for their ability to preserve the labile β-lactam ring of penicillins and cephalosporins while catalyzing the selective hydrolysis and/or synthesis of their relatively stable amide bond of their side chains [18], [19]. These enzymes are members of the N-terminal nucleophile (Ntn) hydrolase superfamily, which is characterized by a common catalytic N-terminal nucleophile [20]. They are also capable of effective and enantioselective acylation of amino compounds in aqueous medium and can be used for preparation of individual enantiomers of primary amines, amino alcohols, non-conventional amino acids and aminonitriles [21]–[28].

Taking into account their biotechnological potential, penicillin acylases have been immobilized to create robust biocatalysts with improved stability over a broad range of reaction conditions including ones in organic solvents. The major results were achieved using different immobilization techniques [29] – incorporation of the enzyme into soluble-insoluble polyelectrolyte complexes [30] or lipid biocomposite [31], chemical cross-linking of enzyme crystals (CLEC) [32] or enzyme aggregates (CLEA) [33], [34], covalent binding to epoxy-activated acrylic carriers [35], epoxy-Sepabeads [36], [37], and adsorption on Celite rods [38]. Stabilization of penicillin acylase by addition of co-solvents (salts, sugars and polyols) has also been reported [39] outlining the important role of hydrophobic interactions in maintaining the protein structure. Immobilization techniques in general provided good operational stability of the biocatalysts but led to a loss of the catalytic activity of the native enzyme. While meeting the industrial requirements, these results tell little about the fundamental protein stabilization mechanisms and structure-function relationship of the penicillin acylase family. A rare example of engineering the native protein stability implemented random mutagenesis to study the influence of surface residues on the alkaline stability of *E. coli* penicillin acylase [40] and resulted in a two-fold increase of the enzyme's half-life period. In a different study a structure-driven computational approach was applied to design penicillin acylase mutants with up to three-fold increase of the thermal stability compared to the wild type [41].

The inactivation kinetics of penicillin acylase from *E. coli* has been studied experimentally in a wide pH range [42]. Very high stability in neutral solutions and rapid inactivation in acidic and alkaline environment was shown. Analysis of kinetic data suggested that the stable and functional protein conformation was maintained by several ionizable residues whose positions in the protein structure were not determined.

While little research has been performed to investigate the basis of pH-dependent denaturation, several stabilization strategies have been proposed for different enzymes. Random mutagenesis combined with screening and selection for the desired phenotype to mimic the Darwinian process (directed evolution) was implemented to produce protein variants with improved pH-stability [43]–[47]. It was further suggested to apply the mutagenesis only to the charged residues located on the protein surface that seemed most likely to lead to the improvement [40], [48]. However, the total number of possible mutations in a protein structure is astronomical and, therefore, stochastic approaches are highly resource-demanding and time-consuming. They require very large mutant libraries as well as efficient screening and selection techniques while still being able to scan only a small fraction of the sequence space. Furthermore, such stochastic approaches are hampered by a high number of deleterious mutations and low number of functionally beneficial phenotypes.

Rapid development of computational technologies as well as availability of quickly increasing genomic/structural databases have diminished the need for unguided evolutionary stochastic approaches and screening of large random libraries. Instead, remarkable progress was achieved by employing comparative structural analysis on M-proteases and alkaline cellulases K with different pH-optimum [49], [50]. These studies concluded that alkaline adaptation is accompanied by a decreased number of negatively charged amino acids and lysine residues and an increased number of arginine and neutral hydrophilic residues (histidine, asparagine and glutamine). Electrostatic interactions of the charged residues were suggested as a key factor of adaptation to extreme pH. Consequently, substitution of basic by acidic residues was used to improve the charge balance and stability at low pH, and vice versa [51]–[54]. It was also established that asparagines and glutamines deamidate in alkaline medium leading to destabilization of the protein structure [55] and mutation of these residues was suggested to improve stability at extreme alkaline conditions [56], [57]. Finally, alteration of pKa values of the key residues – either manually selected [58] or known to be catalytically important [59] – was implemented to design pH-stability profiles. While all these empirical studies suggest a more rational way of engineering pH-stability, they do not provide a clear strategy for selection of the relevant residues to be mutated.

In an alternative approach, the free energies of unfolding of a wild-type protein and its mutants were compared under user-defined environmental conditions to perform wide-scale *in silico* redesign of protein stability. Such methods, however, perform with moderate accuracy and more reliable tools yet have to be developed [60], [61].

The studies discussed above have shown that the pH-dependent protein stability can be modulated by introducing amino acid residues with ionizable side-chains. It is, however, still debated if charged residues on the surface or in the protein core are more important for stability. On the one hand, strong charge-charge interactions on the protein surface stabilize the folded state at neutral pH [62]. On the other hand, a number of mutagenic analyses have shown that electrostatic interactions on the protein surface contribute very little to protein stability suggesting that buried core residues are more important to maintain protein structure [63], [64]. In any case deprotonation/protonation of the ionizable residues at high or low pH is an important factor for protein stability. Consequently, to design pH-stability in a rational way one should evaluate the role of the interactions of ionizable residues, especially those whose side-chains are buried.

In this work bioinformatic analysis was applied to identify the subfamily-specific positions responsible for the function and stability of Ntn-hydrolases. Molecular modeling was used to study the pH-induced unfolding of penicillin acylase from *Escherichia coli* in aqueous medium at alkaline conditions. A buried interaction network has been identified, the collapse of which at alkaline pH leads to destabilization of the native protein conformation. Furthermore, the bioinformatic analysis of homologous Ntn-hydrolases with different pH stability was used to select the hotspot for mutagenesis that can lead to improved protein stability. The computationally predicted mutant was tested experimentally and showed more than 9-fold stabilization under alkaline conditions.

## Results

### 1. Molecular modeling of the pH-induced destabilization

Penicillin acylase (EC 3.5.1.11) from *Escherichia coli* (*Ec*PA) is a heterodimer that contains two monomer chains referred to as α and β, consisting of 209 and 557 amino acid residues, respectively. The two chains are closely interlaced and form a pyramidal structure with the active site located at the bottom of a deep cone-shaped cavity [65]. According to the CATH classification [66], the functional conformation of *Ec*PA contains 5 structural domains – two mainly alpha-helical domains of the α-chain (A1: α3-149 and A2: α150-179) and three domains of the β-chain. The latter include the catalytically active αββα-core structure B1, which, in itself, consists of three sub-domains discretely placed in the sequence (B1-1: β1-72; B1-2: β146-290; and B1-3: β452-536), mainly beta-sheet B2 (β73-145) and mainly alpha-helical B3 (β291-451). The B1 domain is the central domain in the *Ec*PA structure and represents the characteristic four-layer “sandwich” fold of all Ntn-hydrolases, which is composed of two antiparallel β-sheets covered by a layer of α-helixes on each side [67]. This domain, which forms either covalent or non-covalent bonds with all other domains, constitutes the binding site cavity that contains catalytically important residues, and is highly important for the biological function of the enzyme.

A recently developed bioinformatic analysis method [68] was applied to the superfamily of Ntn-hydrolases to identify subfamily-specific positions (SSPs) that are conserved within each functional subfamily, but are different between the subfamilies (see Methods, section 5). Such SSPs are supposed to be responsible for functional discrimination among homologs and thus can be used as hotspots for rational design of functional properties of the enzyme [69]. It has been recently shown that pH-induced denaturation of the penicillin acylase leads to irreversible unfolding of the protein dimer to disordered polypeptide chains [70]. The role of subfamily-specific positions in the pH-induced inactivation of *Ec*PA in neutral and alkaline media was further studied using molecular modeling simulations [71]–[75].

**Figure 1:**
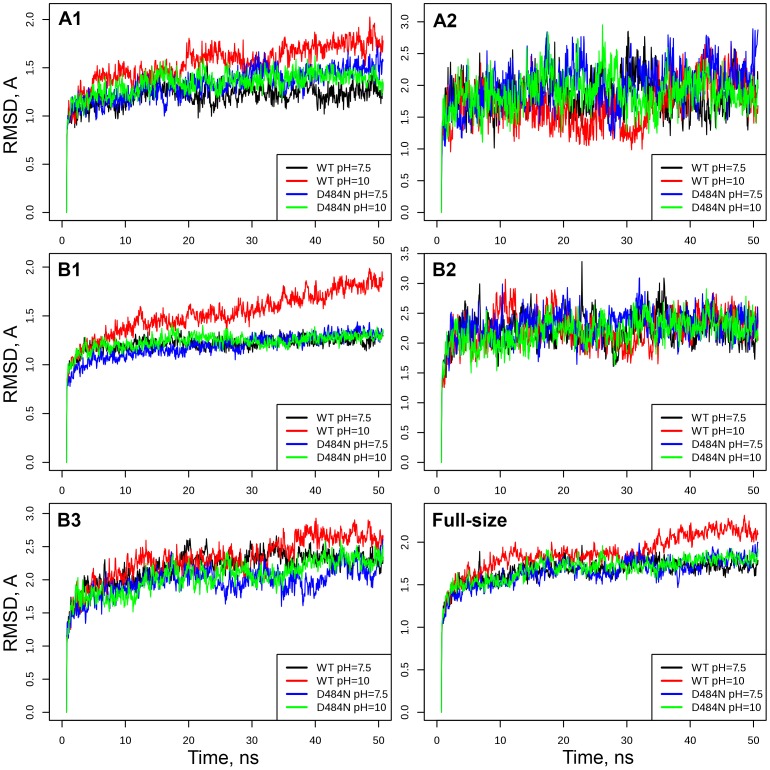
Root mean square deviation (RMSD) of different structural domains during 50 ns MD simulations. Results are shown for *Ec*PA and its Dβ484N mutant at different pH. Each curve is averaged over three independent MD trajectories.

The root mean square deviation (RMSD) of the protein backbone atoms from the initial structure was used as a measure of structural mobility (see Methods, section 1). At neutral pH no global conformational changes were observed in the *Ec*PA. Domains A2, B2 and B3 showed the largest deviation from the initial structure, but did not have a tendency for further destabilization. Domains A1 and B1 remained more consistent during the simulations (Fig. 1). The root mean square fluctuation (RMSF) of C_α_ atoms was used to characterize the structural flexibility and was in agreement with the RMSD data (Fig. S1). All domains with higher RMSF (A2, B2 and B3) also showed larger deviation from the initial conformation, as evidenced by higher RMSD, suggesting that structural flexibility contributes to conformational mobility of the *Ec*PA. Residues within the domain B1 had the smallest average RMSF.

Further comparative analysis of the conformational mobility of *Ec*PA revealed significant structural destabilization at alkaline pH. Under these conditions the domain B1 underwent the largest shift from its native structure, as indicated by the ascending RMSD trend (Table 1). RMSF of C_α_ atoms at alkaline pH showed only slight increment compared to the neutral conditions. Nevertheless, we can note that residues of the domain B1, especially the subdomain B1-3, showed the largest increase of fluctuation that can be explained by a collapse of crucial interactions (Table S1).

**Table 1:** Structural stability of the wild type *Ec*PA at pH 7.5 and 10.0.

Domain	N_res_	RMSD (pH 7.5)	RMSD (pH 10.0)	Δ_RMSD_	*f*
Total	766	1.72±0.13	2.14±0.16	0.24±0.15	0.082
A1	147	1.25±0.19	1.75±0.19	0.28±0.15	0.069
A2	30	1.78±0.50	2.07±0.66	0.07±0.13	0.426
B1	302	1.26±0.13	1.80±0.13	0.37±0.12	0.004
B2	73	2.24±0.37	2.43±0.45	0.05±0.07	0.433
B3	161	2.31±0.43	2.66±0.29	0.20±0.28	0.283

Root mean square deviation (RMSD) for each enzyme variant has been averaged over three independent MD runs. The mean and standard deviation are shown in angstroms. N_res_ – number of amino acid residues in a structural domain. Δ_RMSD_  =  RMSD_pH10_ – RMSD_pH7.5_ and *f*  =  *f*(RMSD_pH7.5_ ≥ RMSD_pH10_) were calculated as explained in Methods, section 2. Lower *f* values indicate a significant destabilizing effect of the alkaline pH compared to neutral conditions while Δ_RMSD_ estimates the degree of destabilization.

The domain B1 involved seven charged residues, which can be in different ionization states at neutral and alkaline environment. These residues were further analyzed to identify the cause of the observed structural changes. Five of these residues that were found to be exposed and the buried residue Aspβ73 located in the Ca^2+^-binding site did not show significant pH-sensitive fluctuations during the MD simulations. In contrast, the core amino acid Gluβ482 – one of the most significant subfamily-specific positions (Table 2) – appeared to be crucial in maintaining the active enzyme conformation.

**Table 2:** Subfamily-specific positions in the chain B of the Ntn-hydrolase superfamily.

Specificity score	Position in *Ec*PA	Subfamily 1: *Ec*PA and close relatives (20 sequences)	Subfamily 2: *Af*PA and close relatives (10 sequences)	Subfamily 3: *Ab*PA and close relatives (10 sequences)	Subfamily 4: *Bm*PA and close relatives (4 sequences)	Subfamily 5: GAs (48 sequences)	Subfamily 6: AHLs (PvdQ) (138 sequences)
1.894	Aspβ484	N(50%) D(50%)	N(80%) D(20%)	F(90%) Y(10%)	H(100%)	A(75%) S(8%)	Q(94%)
1.885	Asnβ20	N(100%)	N(100%)	N(100%)	S(100%)	A(65%) Q(21%)	A(60%) G(40%)
1.582	Gluβ482	E(100%)	E(80%) Q(20%)	A(90%) L(10%)	E (100%)	Y(38%) W(33%) F(29%)	Y(86%) F(9%)

Positions are ranked in a declined statistical significance (see [68] for details). PA – penicillin acylases, GAs – glutaryl-7-aminocephalosporanic acid acylases, AHLs (PvdQ) – N-acyl homoserine lactone acylases PvdQ. The most frequently occurring amino acids are shown for every subfamily.

**Figure 2:**
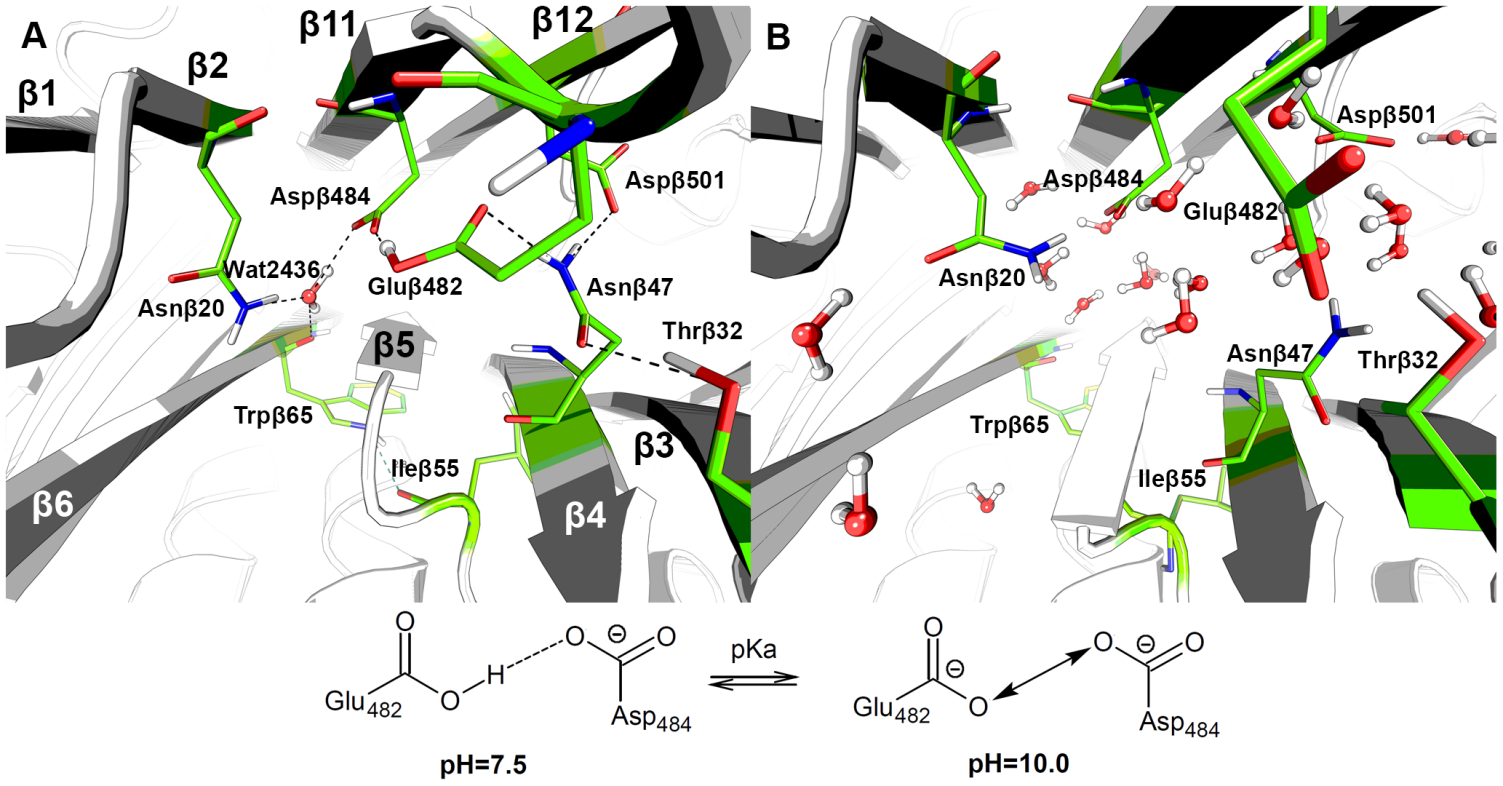
The network of hydrogen bonding interactions between the two β-layers of the αββα-core B1 domain of *Ec*PA is present at pH 7.5 (A) and collapses at pH 10.0 (B).

Gluβ482 is located in a loop close to the strand β11 of the B1 domain (secondary structure numbering is given as in [67]) in close proximity to Pheβ57 and Aspβ484, which leads to an unusually high pKa value (see Methods, section 4). Molecular modeling has shown that at neutral pH the Gluβ482 is protonated, its side-chain oxygen is 2.5 Å away from the side-chain oxygen of Aspβ484 (strand β11), and the two carboxyl groups form a hydrogen bond. The existence of carboxyl-carboxylate pairs and their role in the maintenance of the protein structure has been previously discussed [76], [77]. At neutral pH one of the side-chains is protonated and the corresponding proton is shared between both carboxyl groups, which results in the formation of a hydrogen bond. In this case, at pH 7.5 the hydrogen bond between Gluβ482 and Aspβ484 is preserved in 90–98% of the unconstrained runs of the molecular dynamic trajectories and participates in a network of stabilizing interactions (Fig. 2A). Gluβ482 forms a second hydrogen bond with the side-chain of Asnβ47 (strand β4), which in turn interacts with the side-chains of Thrβ32 (β3) and Aspβ501 (β12). At the same time Aspβ484 forms a hydrogen bond with a buried water molecule Wat2436. Buried solvent molecules usually form functionally and structurally important hydrogen bonds [78]. In the penicillin acylase structure, Wat2436 is involved in three interactions: it acts as a hydrogen bond acceptor for Aspβ484, a hydrogen bond donor to the side-chain of Asnβ20 (β2) and to the main-chain of Trpβ65 (β6). The side-chain of Trpβ65, in turn, is involved in the formation of a hydrogen bond with the main-chain Ileβ55 (a loop between β4 and β5).

At alkaline pH, both carboxylates – Gluβ482 and Aspβ484 – become negatively charged, which causes a repulsion of the side-chains followed by a collapse of the observed hydrogen bond network. While the time-evolution analysis indicates that secondary structure remains intact in general (Fig. S2, S3, S4, S5), the strands β4-β6 shift towards the underlying helices α1 and α2. Disordering of the side-chains leads to a less compact structure that is easily penetrated by solvent molecules – they fill newly formed cavities and expand them; the globule, therefore, starts to “swell” (Fig. 2B). Further development can lead to the formation of an intermediate between folded and unfolded protein states – a so-called “molten globule” [79].

The identified stabilizing interactions connect two antiparallel β-sheets of the αββα-core domain B1 – β1-β2-β11-β12 and β6-β5-β4-β3. The hydrogen bond between Gluβ482 and Aspβ484 at neutral pH is the cornerstone of this buried network whose collapse at alkaline pH launches detrimental changes in the structure and finally leads to the enzyme's inactivation. It can be noted that strands β1-6 belong to the B1-1 subdomain, while β11 and β12 belong to the B1-3 subdomain. This fact explains the destabilizing effect of the alkaline pH on the domain B1 that was observed during the molecular dynamics simulations (Fig.1).

The αββα-core domain is common for the entire Ntn-hydrolase superfamily [67]. The key residues involved – Asnβ20, Gluβ482 and Aspβ484 – are among the most significant subfamily-specific positions in the Ntn-hydrolases (Table 2) and thus the closest evolutionary relatives of penicillin acylases were further studied to reveal whether variation of amino acid residues at selected subfamily-specific positions is related to different stability of these enzymes in alkaline medium.

### 2. Structural analysis of Ntn-hydrolases

*Ec*PA was compared to other Ntn-hydrolases with known three-dimensional structures – penicillin acylases from *Providencia rettgeri* (*Pr*PA) and *Alcaligenes faecalis* (*Af*PA), glutarylamidases from *Pseudomonas sp*. (*Ps*GA) and *Brevundimonas diminuta* (*Bd*GA), and N-acyl homoserine lactone acylase PvdQ from *Pseudomonas aeruginosa* (*Pa*AHLA). The subfamily-specific positions Asnβ20, Gluβ482, and Aspβ484 in *Ec*PA and in the related enzymes were shown to stabilize the interactions between two antiparallel β-sheets of the αββα-core domain. Such interactions typically involve a dense hydrogen bond network with buried solvent molecules that can have different localization in the structure. These results suggest that the outlined hydrogen bond network that involves subfamily-specific positions is highly important for the stability of all Ntn-hydrolases.

**Figure 3:**
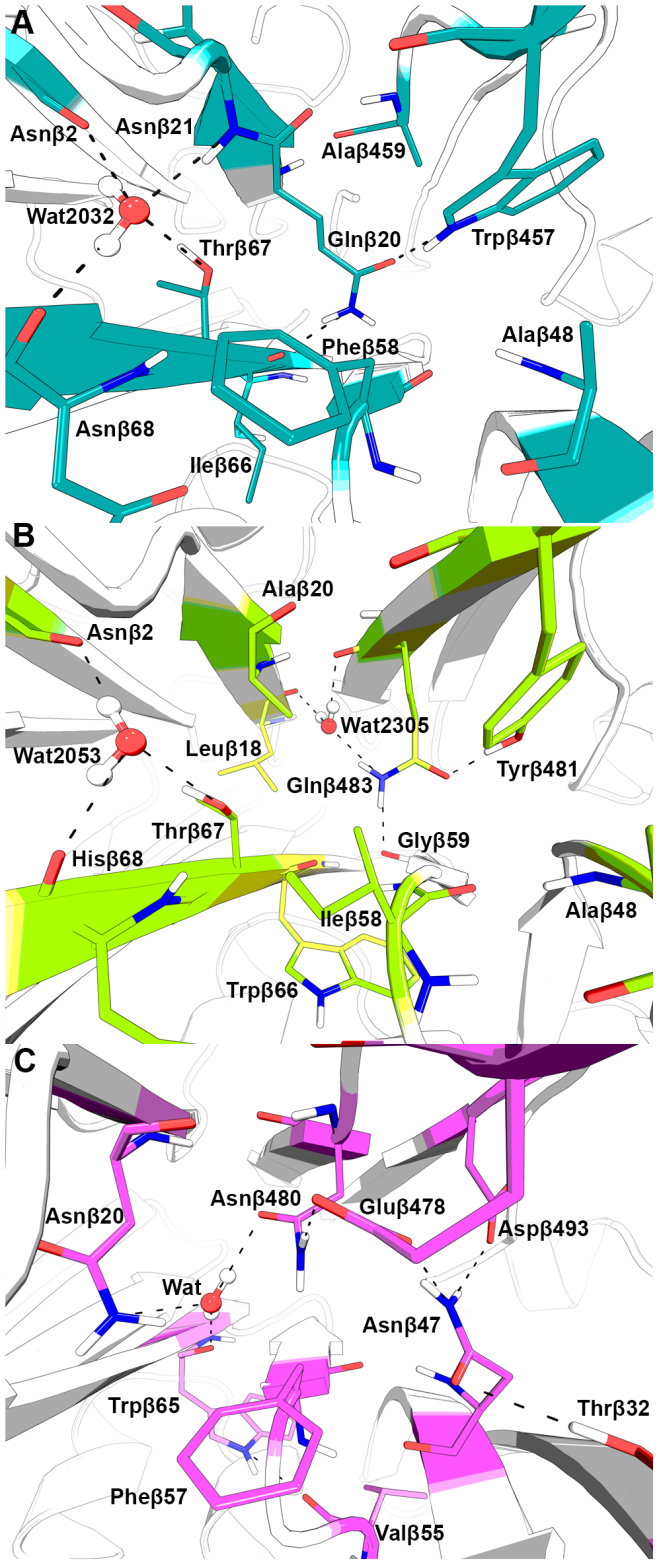
The network of hydrogen bonding interactions between the two β-layers of the αββα-core B1 domain in Ntn-hydrolases. (A) Glutarylamidase from *Pseudomonas sp*., (B) N-acyl homoserine lactone acylase PvdQ from *Pseudomonas aeruginosa*, and (C) penicillin acylase from *Alcaligenes faecalis*.

In *Ps*GA the subfamily-specific positions of Asnβ20, Gluβ482 and Aspβ484 of *Ec*PA are occupied by Glnβ20, Trpβ457, and Alaβ459, respectively (Fig. 3A). While some positions are substituted by residues with different physicochemical properties, the subfamily-specific positions nevertheless remain involved in a similar network of stabilizing interactions between the β1-β2-β11-β12 and β6-β5-β4-β3 sheets. A buried solvent molecule Wat2032 participates in multiple interactions – it can be a hydrogen bond donor to the main-chain of Asnβ2 (strand β1) and Asnβ68 (β6), and a hydrogen bond acceptor from the main-chain of Asnβ21 (β2) and the side-chain of Thrβ67 (β6). It should be noted that Wat2032 in *Ps*GA has a different structural localization compared to Wat2436 in *Ec*PA and a hydrogen bond between the layers is created by the side-chain of Glnβ20 (β2) with the main-chain of Ileβ66 (β6) and the side-chain of Trpβ457 (β11).

In *Pa*AHLA, the subfamily-specific positions Asnβ20, Gluβ482, and Aspβ484 of *Ec*PA are occupied by Alaβ20, Tyrβ481, and Glnβ483, respectively (Fig. 3B). A buried Wat2053 is located in the structure similar to Wat2032 of glutarylamidases and forms three hydrogen bonds with Asnβ2 (β1), Thrβ67 (β6), and Hisβ68 (β6). Another structural water molecule, Wat2305, was found to be positioned in the same manner as Wat2436 in *Ec*PA. It is a hydrogen bond donor to the main-chain atoms of Leuβ18 (β2) and Glnβ483 (β11) and can serve as a hydrogen bond acceptor from the side-chain of Glnβ483, which, in turn, forms two more bonds with Glyβ59 (β5) and Tyrβ481 (β11).

The outlined subfamily-specific positions are conserved in *Pr*PA and *Ec*PA, whereas in the *Af*PA structure, there is a substitution of Aspβ484 by Asnβ480 (Fig. 3C). The side-chain amide group of Asnβ480 in *Af*PA plays a similar role to the protonated carboxylic group of Gluβ482 in *Ec*PA at neutral pH. However, in contrast to *Ec*PA the hydrogen bond formed by the amide-carboxylate pair in *Af*PA can be preserved at both neutral and alkaline pH. The Gluβ482 and Asnβ480 interaction, therefore, is a crucial element of the buried stabilizing interaction network in *Af*PA that makes this enzyme much more stable in alkaline medium than *Ec*PA [80]. Next, we tested the ability of the corresponding mutation Dβ484N-*Ec*PA to enhance the stability of *Ec*PA in alkaline medium.

### 3. *In silico* modeling of catalytic activity and stability of the wild type enzyme and the Dβ484N-*Ec*PA variant

The B1 domain is highly important for the catalytic function of penicillin acylases. In particular, strand β1 hosts the active site Serβ1 while strand β6 has the oxyanion hole residue Alaβ69. The proposed mechanism of penicillin acylase catalysis consists of a nucleophilic attack of Serβ1 on the substrate's amide bond and the corresponding tetrahedral intermediate is stabilized by interactions with the main-chain peptide bond of Alaβ69 and the side-chain amide of Asnβ241 in the oxyanion hole [20]. As the proposed substitution Dβ484N in the B1 domain could jeopardize the catalytic activity, the organization of the active sites of the wild type and mutant enzymes was studied *in silico*. Enzyme-substrate complexes formed by the wild type *Ec*PA and its Dβ484N variant were modeled and used as starting points of MD simulations. To evaluate the catalytic abilities of the enzyme variants the knowledge-based structural criteria were applied to characterize the productive substrate binding mode and the near-to-attack conformation of the substrate in the active site. Three criteria were used – distance between the γ-oxygen of the catalytic Serβ1 and the substrate carbonyl carbon and distances from the substrate carbonyl oxygen to the main-chain amide of Alaβ69 and to the side-chain amide of Asnβ241 – all limited to at most 3.5 Å (see Methods, section 1). A productive substrate binding mode was observed in both enzyme-substrate complexes formed by the wild type *Ec*PA and its Dβ484N mutant at both pH 7.5 and 10.0 (Table 3). This observation is in agreement with experimental data (see Results, section 4) and confirms that Dβ484N mutation does not influence substrate binding at the active site.

**Table 3:** *In silico* evaluation of catalytic activity of the wild type (WT) *Ec*PA and its Dβ484N mutant.

	pH 7.5, 300K		pH 10.0, 300K	
	Near-to-attack conformation, %	Substrate binding energy, kcal/mol	Near-to-attack conformation, %	Substrate binding energy, kcal/mol
**WT**	84.9±9.6	−9.58±1.01	82.2±5.1	−9.11±1.08
**Dβ484N**	89.5±3.4	−9.26±0.74	87.1±8.1	−9.47±0.70

Data for each enzyme variant have been averaged over three independent MD runs.

The stability of the Dβ484N-*Ec*PA variant at different conditions was studied using molecular modeling. The formation of a hydrogen bond between Asnβ484 and Gluβ482 prevailed in 94–99.5% of the unconstrained MD runs at both pH 7.5 and 10.0. No significant differences in the conformational mobility of the wild type enzyme and the Dβ484N variant were observed at pH 7.5. However, at pH 10.0, a significant stabilizing effect of the Dβ484N mutation was monitored in the αββα-core B1 domain (Table 4). These MD studies have shown that in the Dβ484N-*Ec*PA mutant, a buried hydrogen bond network is preserved in alkaline medium and the two antiparallel β-sheets stay connected within the respective domain preventing destabilization of the protein globule in general.

**Table 4:** Modeling of the wild type (WT) and the mutant (Dβ484N) *Ec*PA stability at pH 10.0.

Domain	N_res_	RMSD (WT)	RMSD (Dβ484N)	Δ_RMSD_	*f*
Total	766	2.14±0.16	1.78±0.15	−0.17±0.14	0.138
A1	147	1.75±0.19	1.36±0.19	−0.20±0.16	0.164
A2	30	2.07±0.66	1.90±0.59	−0.05±0.22	0.446
B1	302	1.80±0.13	1.29±0.09	−0.34±0.07	0.003
B2	73	2.43±0.45	2.31±0.35	−0.02±0.06	0.463
B3	161	2.66±0.29	2.32±0.43	−0.18±0.26	0.290

Root mean square deviation (RMSD) for each enzyme variant has been averaged over three independent MD runs. The mean and standard deviation are shown in angstroms. N_res_ – number of amino acid residues in a structural unit. Δ_RMSD_  =  RMSD_Dβ484N_ – RMSD_WT_ and *f*  =  *f*(RMSD_Dβ484N_ ≥ RMSD_WT_) were calculated as explained in Methods, section 2. Lower *f* values indicate a significant stabilizing effect of the mutation compared to the wild type enzyme while Δ_RMSD_ estimates the degree of stabilization.

### 4. Experimental characterization of the Dβ484N variant

The Dβ484N-*Ec*PA variant was purified and studied experimentally. The inactivation rate constants were determined at neutral and alkaline conditions. At pH 7.5 the wild type enzyme and the variant showed comparable inactivation rates, however, at pH 10.0 the Dβ484N mutant was much more stable compared to the wild type *Ec*PA (Table 5). The single mutation led to a 9-fold stabilization at alkaline conditions. The catalytic activity of both enzyme variants was also investigated in neutral and alkaline medium. The initial rates of the enzymatic reaction and the kinetic parameters of the wild type enzyme and the Dβ484N-*Ec*PA mutant were comparable in both cases (pH 7.5 and 10.0). The experimental results were in a good agreement with the computational predictions and showed that the mutation Dβ484N has led to a significant stabilization of the *Ec*PA in alkaline environment without compromising its catalytic function.

**Table 5:** Experimental characterization of the wild type (WT) *Ec*PA and its Dβ484N mutant.

	Catalytic activity						Stability	
	pH 7.5, 25°C			pH 10.0, 25°C			pH 7.5, 50°C	pH 10.0, 25°C
	k_cat_, s^−1^	K_M_, µM	k_cat_/K_M_, µM^−1^ s^−1^	k_cat_, s^−1^	K_M_, µM	k_cat_/KM, µM^−1^ s^-^	k_in_, min^−1^	k_in_, min^−1^
WT	25.0±1.4	25.0±0.8	1.0±0.09	6.4±0.6	156±11	0.04±0.01	0.009±0.001	0.0026±0.0008
Dβ484N	19.5±1.2	14.8±0.5	1.3±0.12	6.5±0.7	110±13	0.06±0.01	0.008±0.001	0.00029±0.00001

## Discussion

Protein stability has been widely studied in the context of industrial needs. It is becoming increasingly evident that the protein ability to tolerate adverse environmental conditions can have important evolutionary implications for the development of new functions. Therefore, understanding the molecular mechanisms by which mutations affect protein stability is an emerging task in protein engineering. New protein functions evolve by the introduction of new phenotypic features by a small number of mutations [81]. As it has been suggested, mutations providing new functions can be destabilizing [7]; such functionally beneficial mutations have to be counterbalanced by additional stabilizing mutations [82], [83]. Bioinformatic analysis of subfamily-specific positions can be used to study stabilization mechanisms in different protein families and develop more effective strategies to implement novel functions.

In this work, bioinformatic analysis and molecular modeling have been used for the first time to identify amino acid residues that are crucial for the pH-stability of penicillin acylase from *E. coli*. A hydrogen bond between the subfamily-specific positions Gluβ482 and Aspβ484 at neutral pH was identified to serve as a basis of the buried stabilizing interaction network connecting two antiparallel β-sheets of the Ntn-hydrolase αββα-core fold. In alkaline medium, both carboxylates – Gluβ482 and Aspβ484 – become negatively charged, which leads to the repulsion of their side-chains and the collapse of the observed hydrogen bond network. Our molecular modeling results show that a hydrogen bond between the mutated residue Dβ484N and Gluβ482 is formed both at neutral and alkaline pH and maintains the observed stabilizing interaction network at alkaline conditions. The Dβ484N-*Ec*PA mutant was produced, studied experimentally and showed an increase of 9-fold in stability at pH 10.0.

Penicillin acylases are key biocatalysts in the industrial production of semisynthetic penicillins and cephalosporins. They are also capable of catalyzing many other potentially valuable reactions such as protection/deprotection of functional groups in peptide synthesis [84] and preparation of enantiomerically pure amines [21]–[23] that are important chiral building blocks for pharmaceutical and agrochemical industries [85]. As primary amines are strong basic compounds, their effective biocatalytic acylation is limited by the low stability and catalytic activity of most enzymes in alkaline solutions. Application of the more stable *Ec*PA variant Dβ484N in alkaline medium and its use as catalyst for preparative enantioselective acylation of amino compounds is under investigation and will be reported elsewhere. The established Dβ484N mutation can also be used to construct templates at rational design of new biocatalysts with improved catalytic properties in the Ntn-hydrolase superfamily.

## Methods

### 1. Modeling of enzyme stability and catalytic activity

Theoretical and computational approaches to study protein structure as a function of solution's acidity have a long history. A number of models have been proposed to perform MD simulations at constant pH taking into account dynamic protonation states of each titratable group in a macromolecule. These ‘constant pH’ methods are thought to allow for sampling over the biologically more meaningful ensemble of conformations at the defined pH. However these calculations are highly demanding due to large number of available protonation states and can still produce significant errors [86], [87]. Therefore in this work we have implemented the traditional notion on pH in MD by setting a constant protonation state of each titratable group at the beginning of the simulation in order to evaluate the internal mobility of a penicillin acylase at different pH. This approach also has some well-known limitations [88], however is simple and relatively fast to implement. In this study it has helped to identify the regions where the unfolding of the protein is initiated at alkaline conditions and demonstrated good correspondence with the experimental data.

The conformational flexibility of the wild type and mutant *Ec*PA were compared at different conditions using molecular modeling. Nanosecond scale MD simulations to study the pH-induced unfolding of *Ec*PA were carried out at two pH values (7.5 and 10.0) and at 373 K temperature. It was shown earlier that increasing the temperature of a molecular dynamics system accelerates protein unfolding without changing its pathway [89]. For each model (free enzyme at defined environmental conditions) three independent MD trajectories with different starting velocities were calculated to improve sampling and collect more data (see Methods, section 3). RMSD of the protein backbone atoms from the initial coordinates was calculated with VMD [90] and used as a measure of structural differences. Pairwise comparisons of last 10 ns of all obtained RMSD time series corresponding to any two different models were made to calculate average Δ_RMSD_ and *f* values (see Methods, section 2). These values were further used to assess the different conformational mobility of *Ec*PA at different conditions. RMSF of Cα atoms relative to the average structure over the last 10 ns was used as an indicator of structural flexibility. Time-evolution analysis of secondary structure was performed using the Timeline plugin in VMD [90].

The ability of the wild type *Ec*PA and the Dβ484N-*Ec*PA variant to form productive enzyme-substrate complexes was studied at two pH values (7.5 and 10.0) and at 300 K temperature. For each model (enzyme-substrate complex at defined environmental conditions) three independent MD trajectories with different starting velocities were calculated (see Methods, section 3). The last 5 ns of the MD trajectories were used to evaluate the near-to-attack conformation of the substrate in the active site using the geometry criteria of the catalytic activity as described earlier [69]. Each frame was used to calculate the distance between the γ-oxygen of the catalytic Serβ1 and the substrate carbonyl carbon as well as distances from the substrate carbonyl oxygen to the backbone nitrogen atom of Alaβ69 and the side-chain nitrogen of the amide group of Asnβ241. The mode of the substrate binding was considered productive if all distances did not exceed 3.5 Å. The binding energy of the substrate in a productive complex was evaluated using the Autodock v.4.2.5.1 scoring function [91].

### 2. Comparison of RMSD time series

We used the following protocol to compare any two MD trajectories. First, the corresponding RMSD time series (2000 frames in each measured with a common frequency) were smoothed by a moving average with window size of 10 frames to reduce the noise [92]. The Dynamic Time Warping (DTW) algorithm [93], [94] was then used to align the two RMSD series by the x-axis (trajectory time). DTW accommodates the cases when elements are similar but out of phase and thus makes one time series to resemble the other as much as possible. Time shifting windows of size 0 (no DTW), 100, 250, 500, 750, 900, and 1000 frames were used. The resulting alignments were used to calculate Δ_RMSD_ and *f* values.

Δ_RMSD_ was calculated for each frame of an alignment as RMSD_MD2_ – RMSD_MD1_ and averaged over all frames of the series. Thus, Δ_RMSD_ between the two MD trajectories can take positive or negative values, depending on the input order. Expectedly, Δ_RMSD_ calculated without DTW were larger in absolute values than those obtained with DTW. At the same time, the results obtained with DTW and various window sizes were not significantly different. By calculating Δ_RMSD_ without DTW (at equal time frames) we, in fact, reduce the two time series to the comparison of only average values, thus losing all the information about particular patterns of MD trajectories. In contrast, DTW provides more accurate and detailed comparison of different trajectories by handling local time shifting.

*f*  =  *f*(RMSD_MD1_ ≥ RMSD_MD2_) is the frequency of frames in aligned trajectories so that RMSD_MD1_ ≥ RMSD_MD2_ and describes to what extent the two curves overlap. *f* values calculated from DTW alignments with various window sizes were not significantly different. Consequently, Δ_RMSD_ and *f* values in Tables 1 and 4 were calculated from DTW with window size of 500 frames and averaged over all pairwise comparisons of MDs between the corresponding models. In both Tables the largest Δ_RMSD_ was observed in the domain B1 with *f* values 0.003–0.004 which indicates significant difference of conformational mobility in corresponding models.

### 3. Molecular dynamics (MD) simulation protocol

Long-time simulations of the enzyme and the enzyme-substrate complexes were carried out using NAMD version 2.9 [95] according to a recently described MD protocol [69]. An input model of a free enzyme or enzyme-substrate complex was prepared as discussed (see Methods, section 4) and parameterized in the Amber force field with AmberTools package version 12 (Case DA, Darden TA, Cheatham TE, Simmerling CL, Wang J, et al., 2012, AMBER 12, University of California, San Francisco). The initial configuration was energy minimized, then all atoms of the protein were constrained using a harmonic energy function and the system was heated to the target temperature. Next, the system was equilibrated and constraints were gradually removed after which a free (unconstrained) run was carried for 50 ns (for free enzyme) or 25 ns (for enzyme-substrate complex). Coordinate snapshots were saved every 5 ps. The simulation of the penicillin acylase model system in a water box (approximately 108000 atoms) for 50.7 ns (equilibration + free run) took 2.7 days on 256 cores of Intel Xeon X5570/Intel Xeon 5670 CPUs. In total, 0.92 µs of MD trajectories were calculated.

### 4. Preparation of the enzyme structure and pKa calculations

The PDB databank entry 1GM9 of *Ec*PA was used as a template for molecular modeling as it has been recently shown that this structure most adequately corresponds to the productive enzyme-substrate complex [96]. Crystallographic solvent molecules and calcium ion were preserved. PDB2PQR v 1.7 was used to add missing hydrogen atoms, resolve steric hindrances and optimize hydrogen bond network [97]. In particular, the program uses a built-in PROPKA heuristic method to calculate pKa values of ionizable residues [98]. Protons were added to the corresponding heavy atoms (OD for Asp, OE for Glu, NZ for Lys, NE for Arg, NE2 or ND1 for His, N for the N-terminal, and O for the C-terminal groups) based on the user-defined environmental pH value: only those residues were protonated that had higher pKa values than the target pH. In three cases, the protonation states of residues from the A1 and B1 domains of *Ec*PA were revised manually. Gluα152 was predicted to have an unusually high pKa value 15.8 as a result of interactions with other carboxylates. This residue is located in the Ca^2+^ binding site and participates in the stabilization of the ion. Both protonation states of Gluα152 were evaluated and no significant impact on the stability was observed (data not shown). The second case was residue Gluβ482. This residue was predicted to have an unusually high pKa 13.8 under the influence of a negatively charged neighboring residue (Aspβ484) and a hydrophobic environment (Pheβ57). To evaluate the impact of Gluβ482 on the protein stability both protonation states were tested and a significant difference was observed that was further investigated in the article. Consequently, in this work Gluβ482 was protonated at pH 7.5 and negatively charged at pH 10.0. Finally, the N-terminal backbone amino group of the active site residue Serβ1 was deprotonated at both pH according to the suggested catalytic mechanism [20].

The PDB databank entry file of penicillin acylase from *Alcaligenes faecalis* (3K3W) contained surprisingly low amount of crystallographic water. Considering the high structural similarity to *Ec*PA and the availability of space between Asnβ20, Trpβ65 and Asnβ480, the water molecule Wat2436 was transferred from 1GM9 to 3K3W and the hydrogen bond network of 3K3W was optimized.

Hydrogen bonds were accepted if the interaction was within 3.5 Å donor-acceptor distance and 145°–180° donor-hydrogen-acceptor angle.

### 5. Bioinformatic analysis of Ntn-hydrolases

We employed the SSM algorithm to query the crystallographic structure 1GM9 of *Ec*PA against the PDB databank [99]. A non-redundant set of five template proteins was retrieved: penicillin acylases from *Providencia rettgeri* (PDB: 1CP9) and *Alcaligenes faecalis* (3K3W), glutarylamidases from *Pseudomonas sp*. (1GK0) and *Brevundimonas diminuta* (1JVZ), and N-acyl homoserine lactone acylase PvdQ from *Pseudomonas aeruginosa* (2WYE). Next, every template protein was independently used as a query for two iterations of PSI-BLAST search [100] against the ‘nr’ database. Sequences sharing less than 0.25 bits-score-per-column with the corresponding template protein were discarded from further analysis [101]. This step was necessary to retain proteins that are not too distantly related and thus should have the same function as the template. At the same time, only one sequence was retained from a cluster with more than 95% pairwise identity to remove redundant sequences. Then, template protein structures were aligned using Matt [102]. The sequence sets were aligned to the corresponding template proteins with T-coffee [103].

Bioinformatic analysis of the subfamily-specific positions responsible for functional diversity within the superfamily of Ntn-hydrolases was performed with Zebra [104]. It has been experimentally shown that different penicillin acylases display remarkable variations in their pH-stability profiles [42], [80]. We analyzed the multiple alignment corresponding to the penicillin acylases with Zebra and determined four functional subfamilies corresponding to close evolutionary relatives of PAs from *E.coli, A.faecalis, A.baumannii*, and *B.megaterium*. These subfamilies were in a good agreement with the experimental data on protein stability. Next, we compared PAs to N-acyl homoserine lactone acylases and glutarylamidases. The list of subfamily-specific positions was ranked by decreasing statistical significance.

### 6. Protein engineering

Wild-type and Dβ484N plasmids were constructed using the modified pBR322 vector carrying the *cat* gene responsible for chloramphenicol resistance. The gene encoding wild type *Ec*PA (analog ATCC 11105) was obtained as described earlier [105]. For cloning and expression of the wild-type and mutant enzymes, *E. coli* TG-1 was used as a host. To introduce Dβ484N mutation into the *Ec*PA gene the QuikChange Site-Directed Mutagenesis (Stratagene, 2010, “QuikChange Site-Directed Mutagenesis Kit,” Mutagenesis, pp. 1–18) was used. The forward primer was 5′- CCGTGGAACAGAAAACAATATGATTGTTTTCTCACC-3′, the reverse primer – 3′-GGTGAGAAAACAATCATATTGTTTTCTGTTCCACGG-5′.

DNA amplification was done using High Fidelity PCR Enzyme Mix (Fermentas) by the following program: 1 cycle –3 min at 95°C; 16 cycles –0.5 min at 95°C, 0.75 min at 60°C, 12 min at 72°C; 1 cycle –5 min at 72°C;. Then, 10 µl of reaction mixture were incubated with 1 µl FastDigest DpnI restrictase at 37°C; for 30 min. Then 50 µl of competent *E. coli* TG-1 cells were transformed by 10 µl of restriction mixture and incubated on ice for 30 min. Heat shock was performed at 42°C; for 1.5 min with the described below incubation on ice for 2 min. A 250 µl LB medium was added to the cells and incubated at 37°C; and 180 rpm for 30 min. A 100 µl of culture fluid was spread on agar plates with 68 mg/l chloramphenicol. After a minimum of 24 hours, 3 clones were transferred into tubes containing 2 ml LB medium with 68 mg/l chloramphenicol and incubated overnight at 37°C and 200 rpm. After that overnight cultures were divided into two parts. The first one was assigned to plasmid preparation (170 mg/l chloramphenicol, 10–12 hours), isolation (GeneJET Plasmid Miniprep Kit, Fermentas) and sequencing (www.evrogen.ru service). The second part was assigned to penicillin acylase expression (0.1 mM IPTG, 15°C, 48 hours, 150 rpm), isolation (osmotic shock), and purification (hydrophobic chromatography).

### 7. Kinetic measurements

Chemicals for experimental research: 2-nitro-5-(phenylacetamido)-benzoic acid (NIPAB) was purchased from Sigma, phenylmethylsulfonyl fluoride (PMSF) from Merck, chloramphenicol (*3% levomycetin*) from Tver pharmaceutical factory, dNTP and IPTG from Helicon.

Kinetic characterization was carried out according to the guidelines established by the European Working Party on Biocatalysis [106]. The penicillin acylase activity was measured spectrophotometrically at 400 nm by determining the release of 2-nitro-5-aminobenzoic acid in the course of NIPAB hydrolysis. The *Ec*PA-catalyzed reaction was performed in a thermostatted reaction vessel at 25°C, 10 mM phosphate buffer pH 7.5, 0.1 M KCl. Absolute concentration of the enzyme's active sites was determined by *Ec*PA titration with a highly specific irreversible inhibitor PMSF as described earlier [107]. The kinetic parameters of the enzymatic hydrolysis (*K*_M_ and *k*_cat_) were determined by initial rate analysis from the experimental dependence of the initial reaction rate on the substrate (NIPAB) concentration (0.5–50.0 µM). The values of *k*_cat_ and *K*_M_ were found by nonlinear regression.

The stability of the wild type *Ec*PA and the Dβ484N-*Ec*PA mutant were investigated at pH 10.0 (10 mM CAPS, 25°C) and pH 7.5 (10 mM KH_2_PO_4_, 50°C). Inactivation followed first-order rate kinetics. The values of the corresponding inactivation constants (k_in_) were determined by interpolation of the experimental data according to the equation A_t_ = A_0_*exp(-k_in_*t), where A_t_ is the residual enzyme activity, A_0_ is the initial activity and t is the time of incubation.

## Supporting Information

Figure S1 Structural fluctuation (RMSF) of *Ec*PA and its Dβ484N mutant at different pH. Each curve is averaged over three independent MD trajectories. Residue numbers are given in an absolute order. Dashed line separates chains α and β, consisting of 209 and 557 amino acid residues, respectively. Residues β482 and β484 are indicated by a red arrow. These positions had one of the lowest RMSF values (∼0.5 Å) which were the same in the wild type enzyme and its mutant at different conditions.
(TIF)

Figure S2 Secondary structure as a function of time, for **wild type** *Ec*PA at **pH 7.5**. The plot is based on a single representative MD, selected from three independent MD runs for the given protein at given conditions. Residue numbers are given in an absolute order. Dashed line separates chains α and β, consisting of 209 and 557 amino acid residues, respectively. Residues β482 and β484 are indicated by a red arrow.
(TIF)

Figure S3 Secondary structure as a function of time, for **wild type** *Ec*PA at **pH 10.0**. The plot is based on a single representative MD, selected from three independent MD runs for the given protein at given conditions. Residue numbers are given in an absolute order. Dashed line separates chains α and β, consisting of 209 and 557 amino acid residues, respectively. Residues β482 and β484 are indicated by a red arrow.
(TIF)

Figure S4 Secondary structure as a function of time, for **Dβ484N mutant** of *Ec*PA at **pH 7.5**. The plot is based on a single representative MD, selected from three independent MD runs for the given protein at given conditions. Residue numbers are given in an absolute order. Dashed line separates chains α and β, consisting of 209 and 557 amino acid residues, respectively. Residues β482 and β484 are indicated by a red arrow.
(TIF)

Figure S5 Secondary structure as a function of time, for **Dβ484N mutant** of *Ec*PA at **pH 10.0**. The plot is based on a single representative MD, selected from three independent MD runs for the given protein at given conditions. Residue numbers are given in an absolute order. Dashed line separates chains α and β, consisting of 209 and 557 amino acid residues, respectively. Residues β482 and β484 are indicated by a red arrow.
(TIF)

Table S1 Structural flexibility (RMSF) of wild type and mutant *Ec*PA at pH 7.5 and 10.0. RMSF for each variant has been averaged over three independent MD runs. The mean and standard deviation are shown in angstroms.
(TIF)
