# Supplementary figures and images for: Computational Design of a pH Stable Enzyme: Understanding Molecular Mechanism of Penicillin Acylase's Adaptation to Alkaline Conditions
Source: PLoS One. 2014 Jun 24;9(6):e100643. doi: 10.1371/journal.pone.0100643 (PMC4069103; doi:10.1371/journal.pone.0100643)

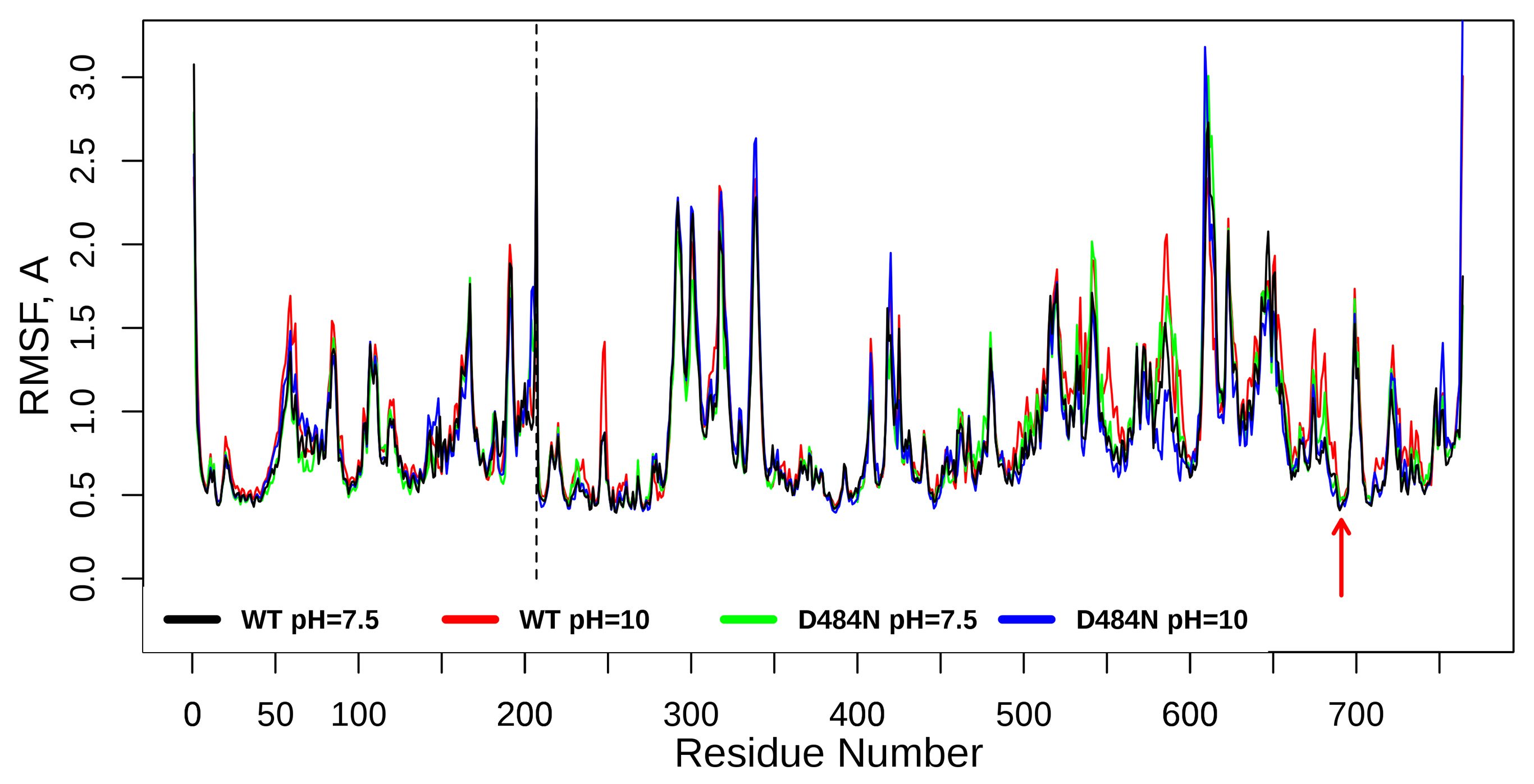

Supplement: Figure S1 — Structural fluctuation (RMSF) of EcPA and its Dβ484N mutant at different pH. Each curve is averaged over three independent MD trajectories. Residue numbers are given in an absolute order. Dashed line separates chains α and β, consisting of 209 and 557 amino acid residues, respectively. Residues β482 and β484 are indicated by a red arrow. These positions had one of the lowest RMSF values (∼0.5 Å) which were the same in the wild type enzyme and its mutant at different conditions. (TIF) [file pone.0100643.s001.tif]

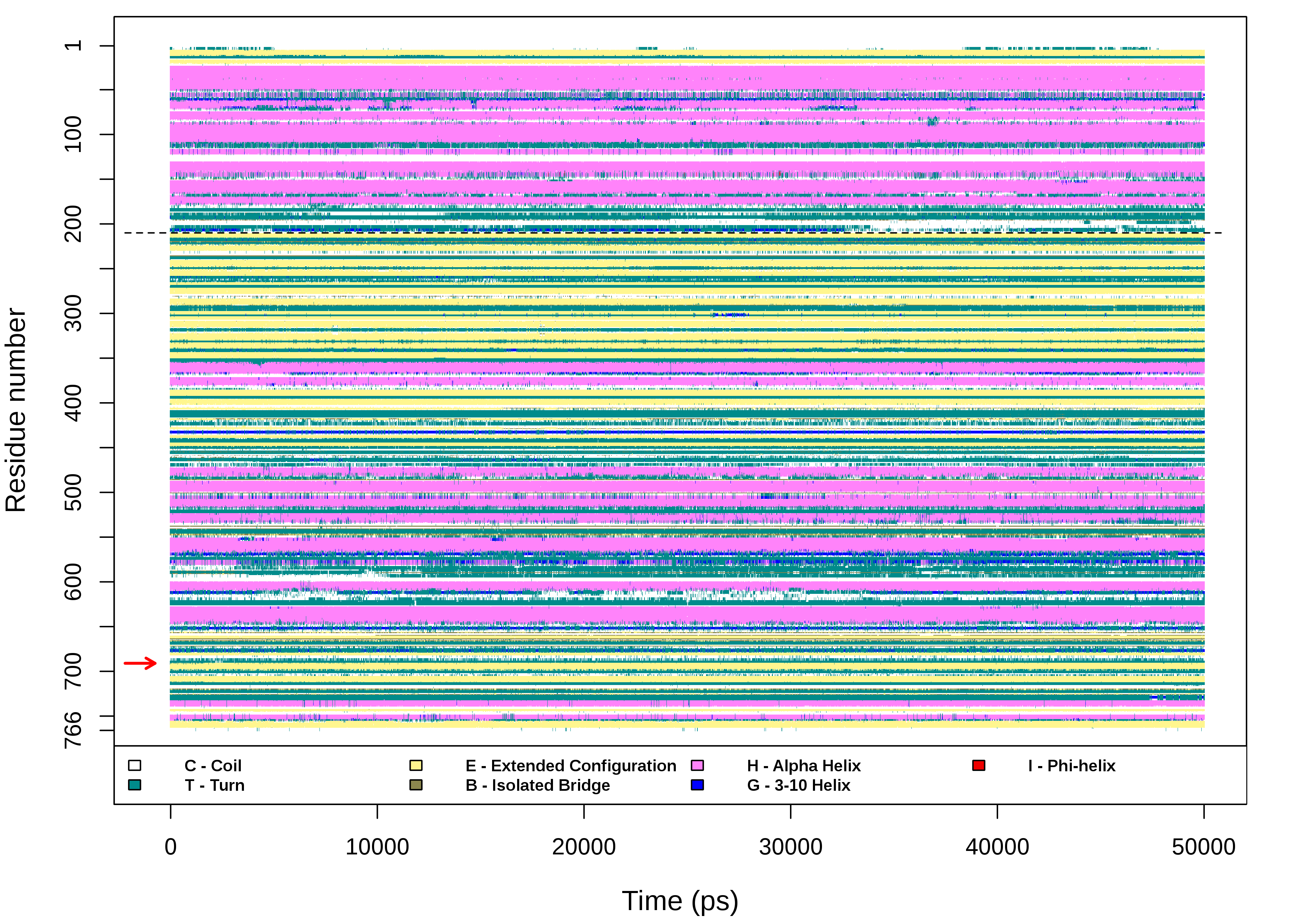

Supplement: Figure S2 — Secondary structure as a function of time, for wild type EcPA at pH 7.5. The plot is based on a single representative MD, selected from three independent MD runs for the given protein at given conditions. Residue numbers are given in an absolute order. Dashed line separates chains α and β, consisting of 209 and 557 amino acid residues, respectively. Residues β482 and β484 are indicated by a red arrow. (TIF) [file pone.0100643.s002.tif]

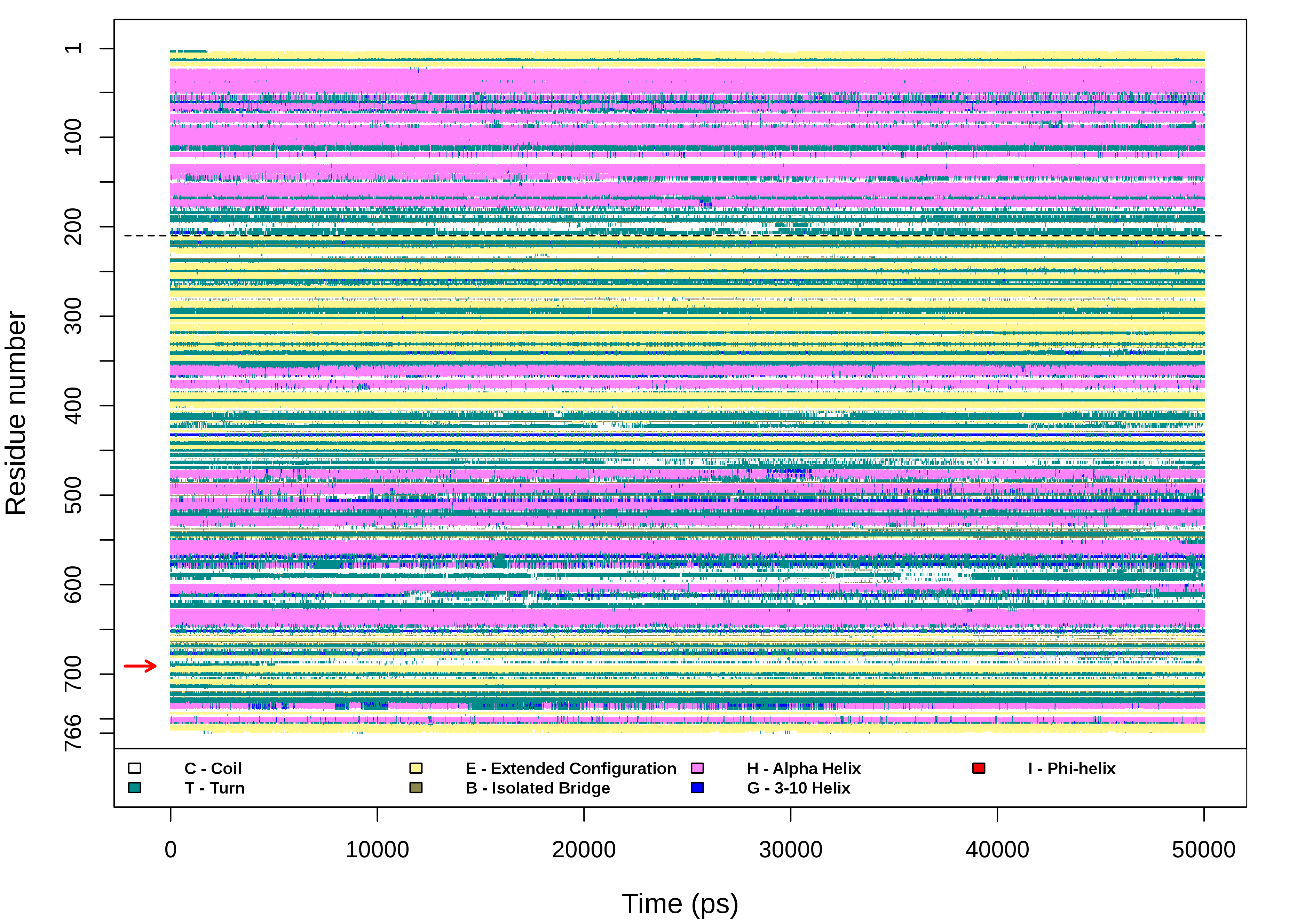

Supplement: Figure S3 — Secondary structure as a function of time, for wild type EcPA at pH 10.0. The plot is based on a single representative MD, selected from three independent MD runs for the given protein at given conditions. Residue numbers are given in an absolute order. Dashed line separates chains α and β, consisting of 209 and 557 amino acid residues, respectively. Residues β482 and β484 are indicated by a red arrow. (TIF) [file pone.0100643.s003.tif]

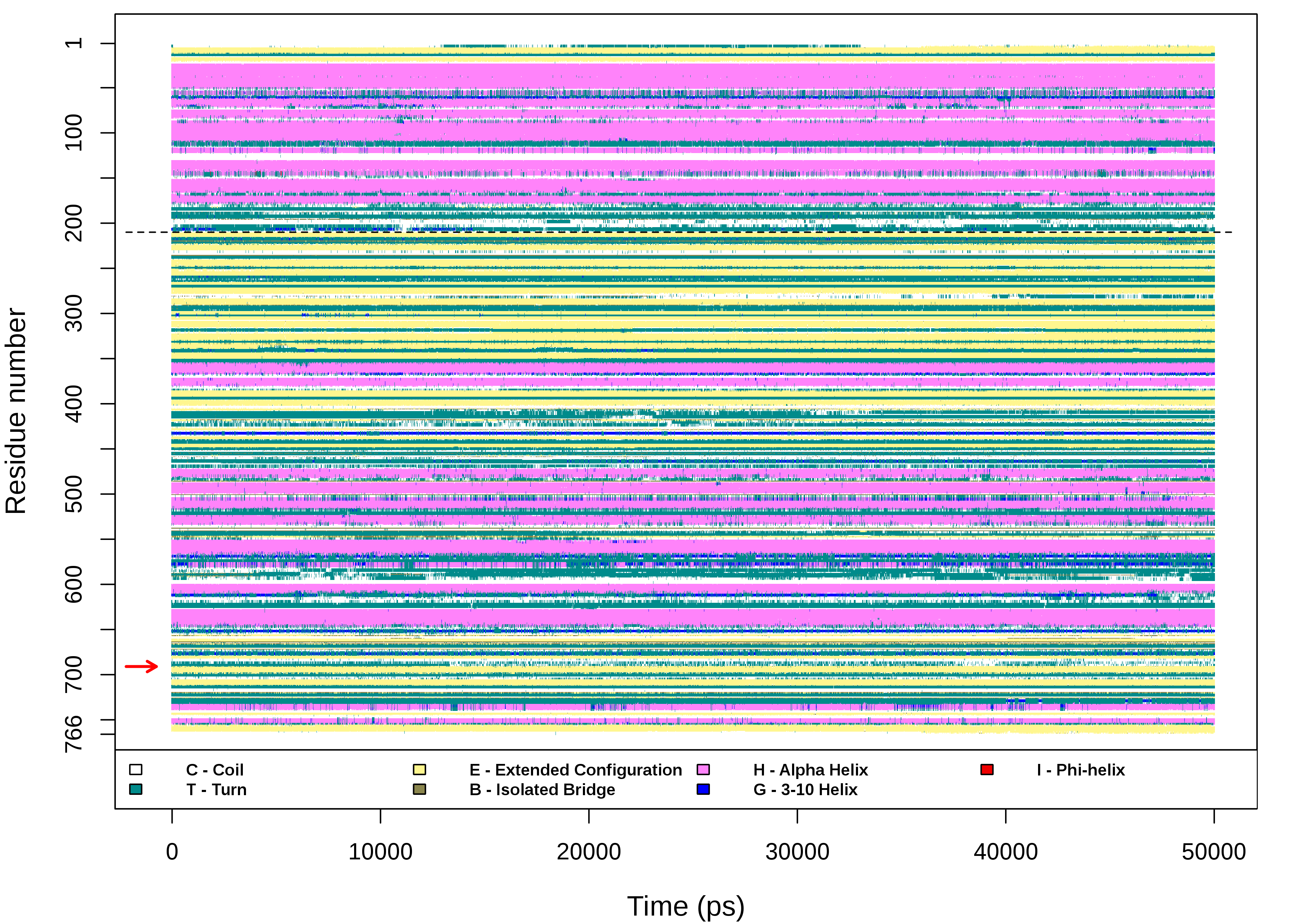

Supplement: Figure S4 — Secondary structure as a function of time, for Dβ484N mutant of EcPA at pH 7.5. The plot is based on a single representative MD, selected from three independent MD runs for the given protein at given conditions. Residue numbers are given in an absolute order. Dashed line separates chains α and β, consisting of 209 and 557 amino acid residues, respectively. Residues β482 and β484 are indicated by a red arrow. (TIF) [file pone.0100643.s004.tif]

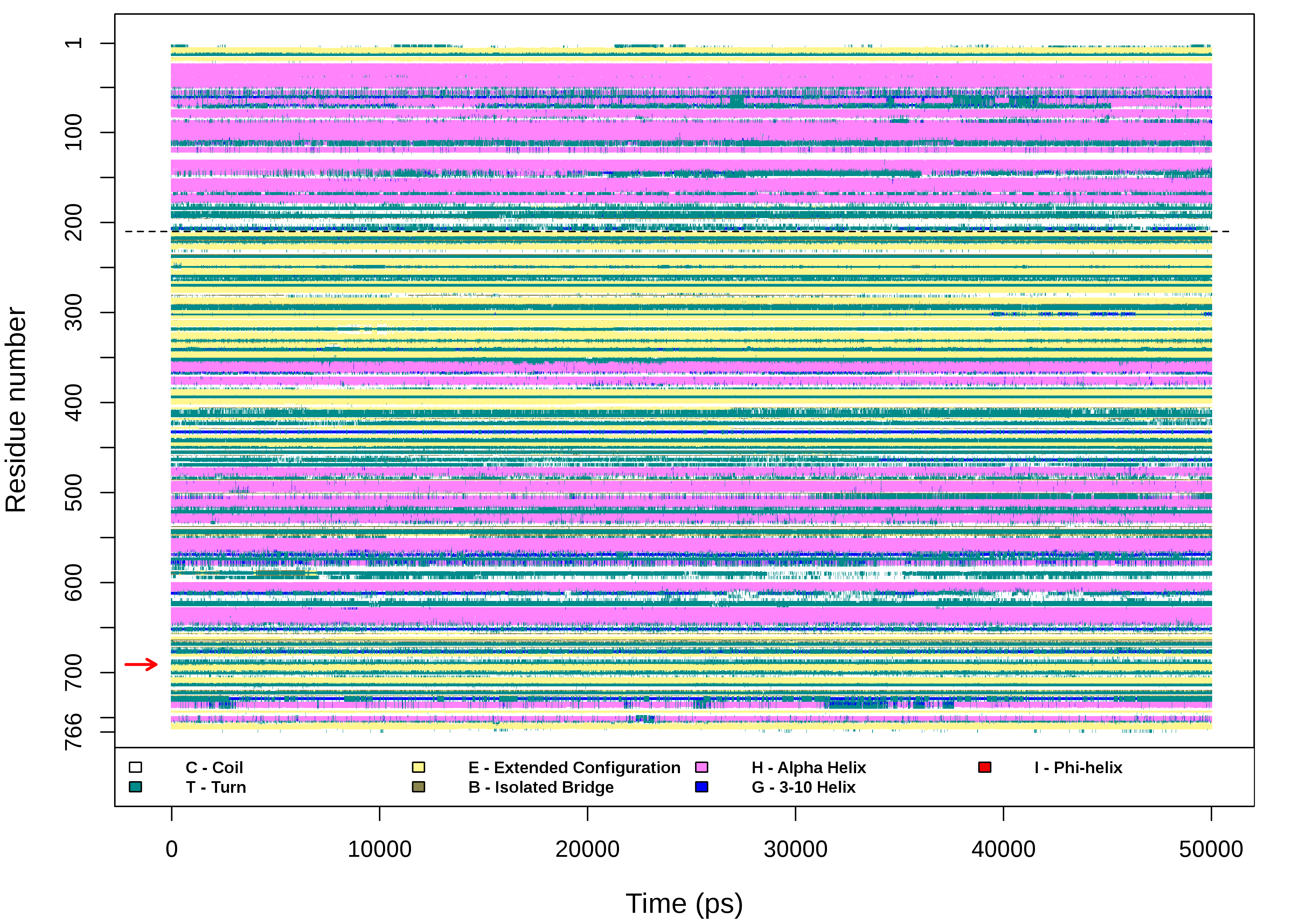

Supplement: Figure S5 — Secondary structure as a function of time, for Dβ484N mutant of EcPA at pH 10.0. The plot is based on a single representative MD, selected from three independent MD runs for the given protein at given conditions. Residue numbers are given in an absolute order. Dashed line separates chains α and β, consisting of 209 and 557 amino acid residues, respectively. Residues β482 and β484 are indicated by a red arrow. (TIF) [file pone.0100643.s005.tif]

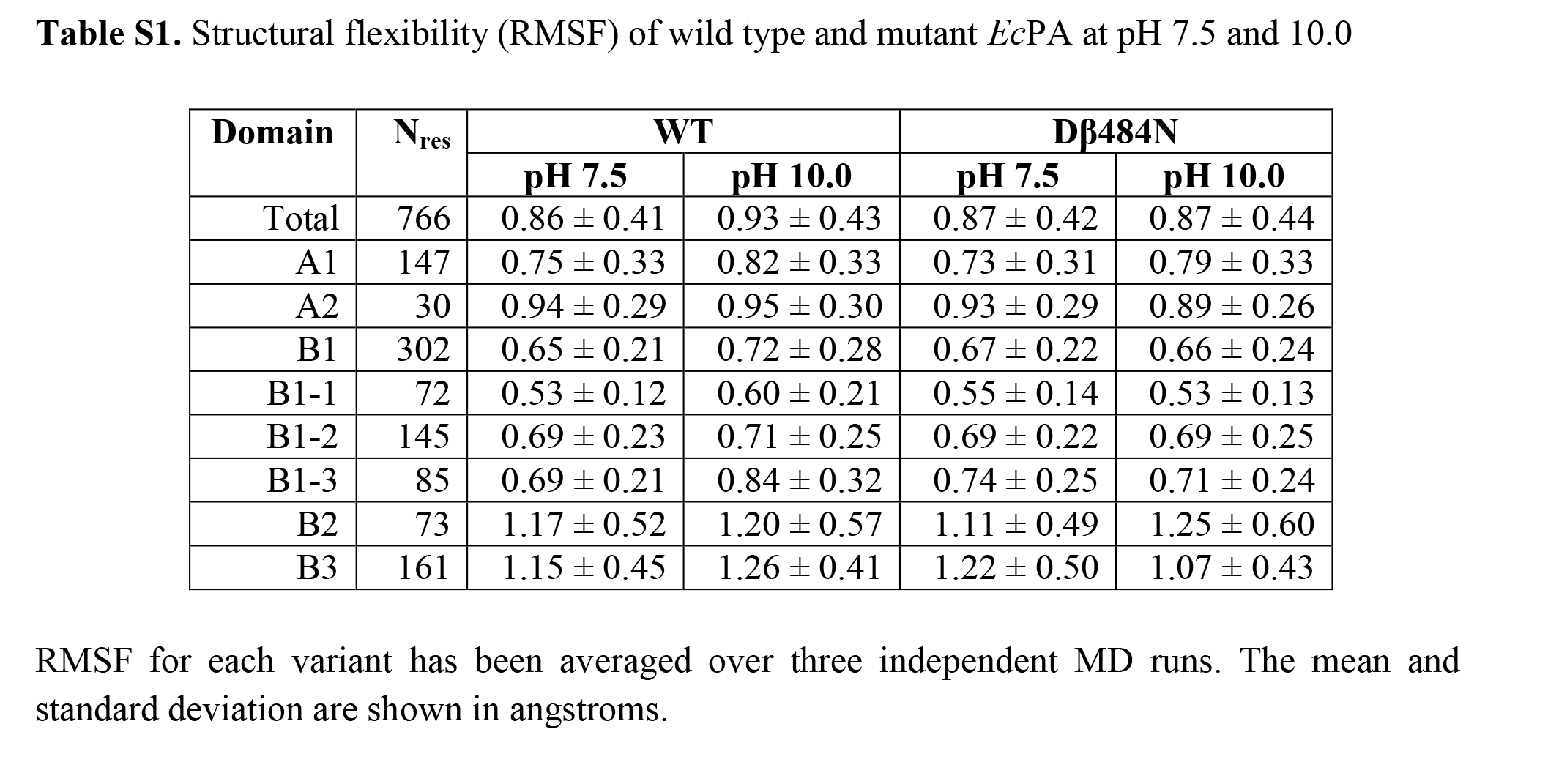

Supplement: Table S1 — Structural flexibility (RMSF) of wild type and mutant EcPA at pH 7.5 and 10.0. RMSF for each variant has been averaged over three independent MD runs. The mean and standard deviation are shown in angstroms. (TIF) [file pone.0100643.s006.tif]
